# Investigation of strained deformed state of variable stiffness rod

**DOI:** 10.1186/2193-1801-3-367

**Published:** 2014-07-19

**Authors:** Sergey Tsarenko, Gennady Ulitin

**Affiliations:** The Strength of Materials Department, Donetsk National Technical University, Donetsk, 83001 Ukraine; The Further Mathematics Department, Donetsk National Technical University, Donetsk, 83001 Ukraine

## Abstract

An equation for bending of a weighable rod with variable transversal stiffness was proposed. On the basis of this analyses the conclusions were drawn about the influence of parameters of construction on values of maximum sag and maximum bending moment. The recommendations for the usage of the simplified model were done. The example of the construction with given parameters for calculation of stiffness and strength according to the represented mathematical models was considered.

Different models of rods are used for investigation of building constructions which are under the influence of longitudinal and transversal loads (Editor Madugula 2002). For example, such models in stability and dynamics problems were used in the works (Yoo 2011; Strommen Einar 2014; Yang 2005). For the first time the differential equation of longitudinal bend of rods of variable section under the influence of point load was considered in the paper (Ostwald 1889). Stability under the influence of distributed longitudinal load for pointed rods was investigated by L. Bairstow (Bairstow L, Jones BM, Thompson BA 1913). Analytical solution of the common problem for the constructions, which are under the influence of longitudinal point and distributed loads, is not found in literature. In practice, when the model of equivalent rod is used, the influence of longitudinal loads on the value of transversal deformation and bending moment is not taken into account or calculated methods are used (Editor Madugula 2002). The accurate definition of the model, taking into account longitudinal and transversal loads, is a new problem.

The purpose of this paper is:

- getting formulas for investigation of strained deformed condition of a rod which is a model of a construction of a lattice tower under the influence of longitudinal and transversal loads;
- valuation of the influence of longitudinal loads on the value of transversal deformation and bending moment.

Mathematical model of elastic weighable rod with variable bending stiffness and length *l* is considered for the study. The axis force *N*_0_ acts on the rod, *p*(*x*) - horizontal distributed load (Figure 1). As an example We take a linear distributed load . Here *h* = *ax* + *h*_1_ is a radius of rigidity of a variable section, *h*_*1,*_*h*_*2*_ are radii rigidity of upper and lower sections, a , *A* is cross sectional area, *E* is elastic modulus.

**Figure 1:**
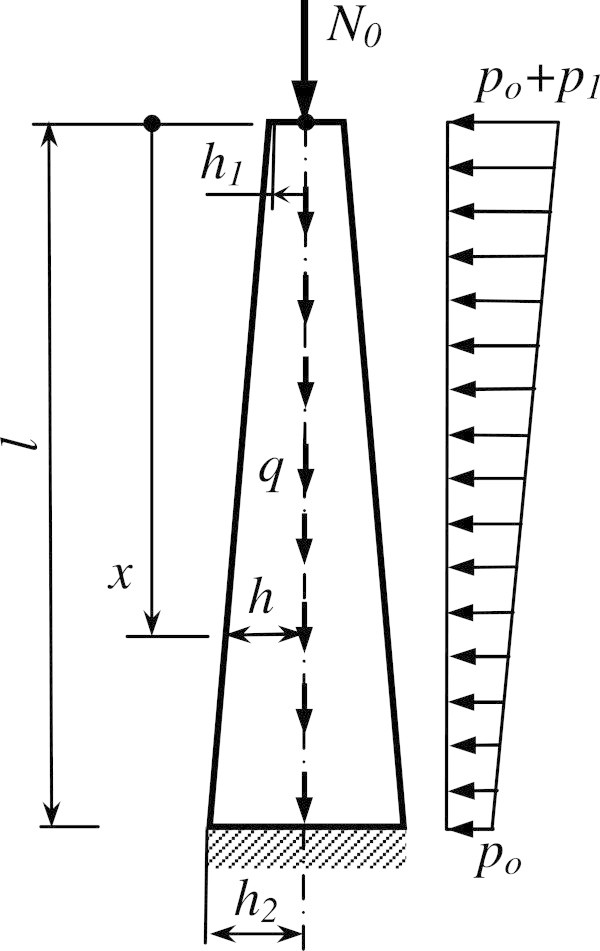
**The calculated scheme of the construction of lattice tower.**

We work out the equation of an element balance of the construction
123

Defining from the equations (1-3) moments *M*(*x*) and transversal forces *Q*(*x*) we arrive at the equation in *y(x)* – horizontal (y-axis) displacement *y*(*x*) -
4

We introduce the equation (4) as
5

We have after integration (5)
6

We make the substitution variables for equation (6) ; where  and . Then it looks like
7

where ,  or .

The inhomogeneous equation (7) corresponds to the homogeneous one
8

Its solution is known (Kamke 1942)
9

We find common solution of inhomogeneous equation (7) by the method of arbitrary constants variation.

The Wronskian of the fundamental system of functions ,  (Ostwald 1910), then , *W* ≠ 0 because *v* is not an integer figure.

After using the arbitrary constants variation method the common solution of the equation (5) looks like
10

where *S*_μ,ν_(*z*) - Lommel functions (Watson 1922).

Lommel functions for problems of stability were used in the papers (Ulitin 2002; Elishakoff and Pellegrini 1988).

Taking into account that  and integrating the formula (10), we get the equation of the bend rod axis
11

where ; .

It is possible to define derivatives  and  from the formula (11) by using recurrent formulae for Bessel and Lommel functions:
1213

After their definition we get the expressions for the moments
14

and transversal forces
15

Using the formulas (10-13) it is possible to calculate stiffness and strength, where constant integrations *С*_1 − 4_ are defined from bordering conditions for fastening or joint of construction parts.

We consider two models (Figure 2) for valuation of the influence of longitudinal loads on the value of transversal deformation and bending moment. We bring in values non-dimensions for these models:  – a moment non-dimension in the base of a rod;  – sag non-dimension of a rod apex;  – weight non-dimension of a construction;  – transversal force non-dimension.

**Figure 2:**
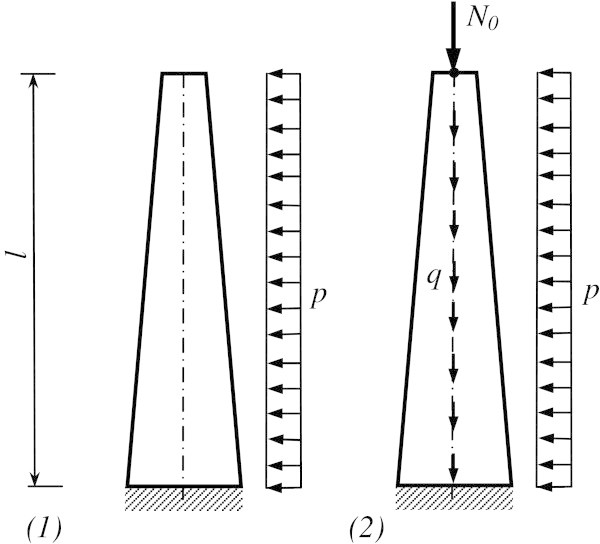
**The calculated models of a construction of a lattice tower: 1 – the model without taking into account longitudinal load; 2 – the model which takes into account longitudinal load.**

We get from formulas (1-3) for the model (1):


We get expressions  and  for the model (2) from formulas (11-12) taking into account *p*_0_ = *p*, *p*_1_ = 0. Constant integrations are defined from bordering conditions
16

Corresponding calculated results of the investigation are represented in Figures 3, 4 and 5.

**Figure 3:**
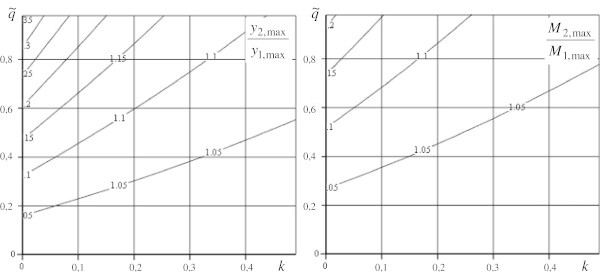
**The ratios of sags and bending moments for models for**  **.**

**Figure 4:**
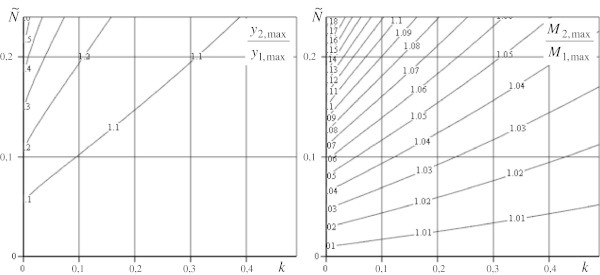
**The ratios of sags and bending moments for models for**  **.**

**Figure 5:**
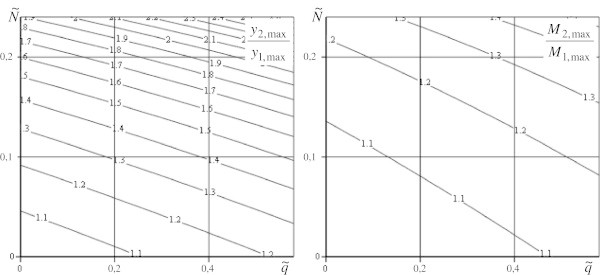
**The ratios of sags and bending moments for models for** ***k*** **=** **10** ^**−** **2**^ **.**

On the bases of the received results we can make the following conclusions:

1. The influence of longitudinal loads on value of sags and bending moments considerably diminish with increasing parameter *k* (Figure 3;Figure 4);
2. We get maximum values of loads for using the model (1) exactness of which is up to 5% for pointed rod (*k* = 001), it is represented in graphics of Figure 5,
  and  for maximum sag definition;
  and  for maximum moment definition.

For example, a boring rig, which represents a rod construction in the form of a square truncated pyramid, has the following parameters: rig height is 53,3 m, width of the low base is10 m, of the upper one is 2 m (*h*_1_ = 1 м; *h*_2_ = 5 м; *k* = 0,25; *a* = 0,075), transversal section area is 351,4 ⋅ 10^− 4^ m^2^, rig weight is 4 ⋅ 10^5^ N (), equipment weight 3,2 ⋅ 10^6^ N (). The ratios of sags and moments form, *y*_2,max_/*y*_1,max_ = 1045; *M*_2,max_/*M*_1,max_ = 1022 for it, so the error of the model (1) use for the definition of sag is 4,5% and of moment in the base is 2,2%.

The graphs in Figures 3, 4 and 5, show that in the common case the longitudinal load influences the calculated parameter values. So, when investigating a strained deformed condition of a structure presented above, it is necessary to use this suggested model or on its base to ground established tolerances in calculated schemes.

The problem, which is under consideration in this paper, is a tower type constructions model under the influence of point load and distributed loads. This model can be used for the investigation of strained deformed condition of constructions, for solving problems of stability and for mathematical description of elements in program modeling complexes on the base of finite elements method.
